# FLASH radiotherapy for the treatment of symptomatic bone metastases in the thorax (FAST-02): protocol for a prospective study of a novel radiotherapy approach

**DOI:** 10.1186/s13014-024-02419-4

**Published:** 2024-03-12

**Authors:** EC Daugherty, Y Zhang, Z Xiao, AE Mascia, M Sertorio, J Woo, C McCann, KJ Russell, RA Sharma, D Khuntia, JD Bradley, CB Simone, JC Breneman, JP Perentesis

**Affiliations:** 1 Department of Radiation Oncology, University of Cincinnati, Cincinnati, OH USA; 2 Cancer and Blood Disease Institute , Cincinnati Children’s Hospital , Cincinnati, OH USA; 3 Department of Radiation Oncology, University of Pennsylvania, Philadelphia, PA USA; 4 Department of Radiation Oncology, New York Proton Center , New York, NY USA; 5 Varian, a Siemens Healthineers Company, Palo Alto, USA

**Keywords:** FLASH radiotherapy, Proton therapy, Thoracic bone metastases, FLASH workflow, Safety, Efficacy, Ultra-high dose rate, Adverse effects, Pain relief

## Abstract

**Background:**

FLASH therapy is a treatment technique in which radiation is delivered at ultra-high dose rates (≥ 40 Gy/s). The first-in-human FAST-01 clinical trial demonstrated the clinical feasibility of proton FLASH in the treatment of extremity bone metastases. The objectives of this investigation are to assess the toxicities of treatment and pain relief in study participants with painful thoracic bone metastases treated with FLASH radiotherapy, as well as workflow metrics in a clinical setting.

**Methods:**

This single-arm clinical trial is being conducted under an FDA investigational device exemption (IDE) approved for 10 patients with 1–3 painful bone metastases in the thorax, excluding bone metastases in the spine. Treatment will be 8 Gy in a single fraction administered at ≥ 40 Gy/s on a FLASH-enabled proton therapy system delivering a single transmission proton beam. Primary study endpoints are efficacy (pain relief) and safety. Patient questionnaires evaluating pain flare at the treatment site will be completed for 10 consecutive days post-RT. Pain response and adverse events (AEs) will be evaluated on the day of treatment and on day 7, day 15, months 1, 2, 3, 6, 9, and 12, and every 6 months thereafter. The outcomes for clinical workflow feasibility are the occurrence of any device issues as well as time on the treatment table.

**Discussion:**

This prospective clinical trial will provide clinical data for evaluating the efficacy and safety of proton FLASH for palliation of bony metastases in the thorax. Positive findings will support the further exploration of FLASH radiation for other clinical indications including patient populations treated with curative intent.

**Registration:**

ClinicalTrials.gov NCT05524064.

## Background

FLASH therapy is an emerging form of radiation therapy (RT) in which radiation is delivered at ultra-high dose rates of at least 40 Gy/s. Compared to RT given at conventional dose rates, FLASH RT has been shown in multiple animal models to cause less injury to normal cells and tissue, while having equal or greater tumor cell killing [1–6]. The mechanism for this protective effect of normal tissues by FLASH is thought to be related, in part, to FLASH resulting in lower levels of toxic oxygen-reactive species in normal tissues as compared to conventional RT [7]​.

There has been one published case report of the use of electron FLASH RT in a human. A single patient with cutaneous T cell lymphoma and extensive prior RT to the skin was treated with a 15 Gy single fraction using electron FLASH RT for a recurrent cutaneous lymphoma lesion. This resulted in a complete response of the lesion with minimal toxicity to the surrounding skin [8]​.

FAST-01, the first prospective clinical trial of FLASH, evaluated the feasibility of proton FLASH therapy for the palliative treatment of painful bone metastases in the extremities [9]. Ten patients were enrolled and treated with an 8 Gy single fraction regimen delivered at FLASH dose rates to 12 metastatic sites. All treatments were delivered uneventfully, and study participant time on table (which includes positioning on the treatment couch, imaging, and FLASH treatment delivery) averaged 15.8 min. Reduction in pain at the treated sites was consistent with published literature using a single 8 Gy fraction of conventional photon RT (RTOG 9714). The overall pain response rate to FLASH treatment at 3 months was 66.7% (50% complete response: 6/12 treated sites, 16.7% partial response: 2 treated sites). In follow-up, 16.73% (2 of 12) of the FLASH treatment sites required additional treatment. Treatment was well tolerated. There were 23 serious adverse events (SAEs) reported with 22 scored as definitely not related and 1 scored as probably not related to the FLASH treatment. Similar to the prior RTOG 9714 trial, acute, mild grade 1 hyperpigmentation in the FLASH study participants was observed, with 4/10 participants affected compared to 16/433 (15-grade 1 and 1-grade 2) skin AEs in RTOG 9714 [10]. The AEs affecting skin are consistent with known side effects of radiation treatment and no unexpected AEs were reported. The results of the FAST-01 pilot study confirm the feasibility of FLASH radiation treatment in a routine clinical setting and support the further exploration of FLASH radiation treatment for other clinical indications [11].

A long-term goal of future clinical trials is to evaluate FLASH RT for the potentially curative treatment of patients with thoracic malignancies. The novel FAST-02 clinical trial, described in this protocol, builds on the FAST-01 experience and is the next step in further assessing the safety and efficacy of FLASH RT in different anatomic sites. FAST-02 was designed to evaluate the safety profile of FLASH RT in lower-risk thoracic clinical presentations as well as the pain response associated with a palliative FLASH RT regimen prior to treating more complex clinical presentations with curative intent.

Patients with painful bone metastases continue to represent an ideal population for a feasibility study of FLASH therapy, because the palliative effects of standard of care RT using single-dose radiation regimens are well understood [11–14]. It is expected that the participants of this study will receive the same pain control benefits as if treated with conventional dose rate photon RT. Normal tissue toxicity is expected to be mild.

## Methods

### Objectives

The objectives of this clinical trial are.

1. To assess toxicities of FLASH treatment (co-primary objective).
2. To assess FLASH efficacy as pain relief at the treated site(s) (co-primary objective).
3. To assess workflow metrics of FLASH RT to the thorax in a clinical setting (secondary objective).

### Study design

In this prospective clinical trial, 10 study participants will undergo FLASH therapy with palliative intent to 1–3 painful bony metastases in eligible treatment sites in the thorax (e.g., ribs, clavicle, scapula, and sternum, but not the spine). As this is an exploratory study, the sample size was approved by the FDA as sufficient to address the objectives. Study activities will take place at the Cincinnati Children’s Hospital Medical Center (CCHMC) Proton Therapy Center with IRB approval and under an FDA Investigational Device Exemption (IDE). The Cincinnati Cancer and Blood Disease Institute Data Safety Monitoring Board (DSMB) will serve as the DSMB for the study.

Patients who meet the initial eligibility requirements and provide informed consent will be enrolled in the study. Study duration includes radiation treatment simulation, radiation treatment planning, treatment delivery (≤ 10 business days from treatment simulation), and post-treatment follow-up. Participants will continue follow-up activities until they withdraw from the trial or their health precludes further participation.

### Study population

Patients will be informed about the study primarily by radiation oncologists and their staff. Patients eligible to enroll are adults undergoing palliative RT for metastases in bones of the thorax, excluding the spine, who meet all of the inclusion criteria and do not meet any of the exclusion criteria.

In addition to the metastatic sites to be treated with FLASH therapy patients may have other bone metastases that may be treated with conventional RT. In the case that there is more than one lesion treated, no overlap of FLASH radiation fields with other FLASH or conventional radiation fields is allowed.

The initial screening process will assess the initial inclusion criteria. After providing informed consent, study participants will undergo CT simulation imaging for the purpose of treatment planning. The treatment planning process will allow assessment of the final inclusion criterion, i.e., whether a clinically acceptable FLASH treatment plan can be designed for their lesion(s).

Participants in this clinical trial may also be enrolled in other studies, provided that those studies do not have an impact on the primary endpoints of this trial. Steroid medication for the treatment of bone pain is optional and at the discretion of the prescribing physician as needed.

### Initial inclusion criteria

- Patient age ≥ 18 years.
- 1–3 painful bone metastasis(-es) requiring treatment in the ribs, clavicles, scapulae, or sternum; if more than 1 metastasis can be treated within the same treatment field, it will be counted as 1 metastatic site for the purpose of trial treatment.
- Bone metastases that can be treated within a field size up to 7.5 cm x 30 cm without overlap of other radiation fields.
- Life expectancy > 6 months (in the judgment of the investigator).
- Patients who are able to comply with the protocol.
- Provision of signed and dated informed consent form.

### Final inclusion criteria

- A clinically acceptable FLASH treatment plan.

### Exclusion criteria

- > 3 painful bone metastases of the thorax requiring palliative RT. If > 1 metastasis can be treated within the same treatment field, it will be counted as one metastatic site for the purpose of trial treatment; patients with > 3 painful bone metastases in the thorax requiring treatment are more likely to have generalized thoracic pain that may confound measurement of pain relief response to treatment.
- Overlap of FLASH radiation fields with any previous or planned radiation fields to the same site.
- Patients with pathologic bone fractures in the treatment field.
- Patients with metal implants in the treatment field (proton range and dosimetry are less certain in the presence of metal).
- Patients with symptomatic pneumonitis at the time of screening, or a history of symptomatic radiation pneumonitis.
- Patients with known contraindications to thoracic radiation.
- Patients who received or will receive cytotoxic chemotherapy and/or any prescribed systemic therapy known to impact tissue response to radiation within 2 weeks prior or 1 week following their planned radiation treatment (concurrent therapies may affect the tissue response to radiation).
- Prior local therapy modality to the treatment site(s) within 2 weeks of study enrollment that, in the judgment of the investigator, might compromise interpretation of pain response.
- Patients with persistent toxicity ( except for alopecia and peripheral neuropathy) grade > 1 from prior systemic therapy that is within the proposed treatment field.
- Patients with pacemakers or other implanted devices at risk of malfunction during RT.
- Patients with any other medical condition or laboratory value that would, at the discretion of the investigator, preclude the patient from participating in this clinical investigation.
- Patients at known risk of enhanced normal tissue sensitivity to RT due to inherited predisposition or documented comorbidity that might lead to hypersensitivity to ionizing radiation.
- Patients enrolled in any other clinical studies that the investigator believes to conflict with this clinical investigation.
- Patients who are pregnant or nursing.

## Intervention

### CT simulation and planning target volume (PTV)

Simulation imaging will be performed as part of the standard of care in order to develop the radiation treatment plan. During the simulation procedure, the subject will be placed on the CT simulator couch in a stable and reproducible position suitable for targeting the metastatic lesion(s). Immobilization devices such as a Vac-LokTM bag (CIVCO Radiotherapy, Coralville, Iowa), are recommended to aid in immobilizing the target site(s) and for reproducing the subject’s positioning at the time of treatment. The CT imaging will be obtained through the areas of interest and used for RT planning.

Patients will undergo respiratory 4DCT imaging unless a 4DCT is not clinically feasible in the judgment of the treating investigator (see below). The 4DCT scan will be acquired under conditions of free breathing to assess the extent of target motion.

- If the maximum target excursion is > 10 mm, an abdominal compression approach will be used to reduce the extent of motion. An additional 4DCT scan of the subject with abdominal compression will then be acquired and used for treatment planning in combination with an Internal Target Volume (ITV) approach due to residual target motion.
- If the subject’s motion cannot be reduced to less than 10 mm, the subject will be withdrawn from the study and replaced.
- If the maximum target excursion is ≤ 10 mm, the existing 4DCT scan will be used for treatment planning (using an ITV approach). No additional motion mitigation strategies (beyond the ITV approach used in planning) are required.

Where a 4DCT is not clinically feasible, a conventional simulation CT scan with the subject in free-breathing will be acquired and used for treatment planning.

Women of childbearing age must have a negative urine or serum pregnancy test prior to CT simulation. If the pregnancy test is positive, the subject will be removed from the study and replaced.

### Treatment planning

The simulation CT images will be electronically transferred to the Eclipse Treatment Planning System v16.5 (Varian Medical Systems, Palo Alto), as per the standard clinical workflow.

For an ITV planning approach based on the 4DCT, the target(s) will be delineated by one of the radiation oncologist investigators who will designate the Gross Tumor Volume/Clinical Target Volume (GTV/CTV). The ITV will be created based on the target (GTV/CTV) locations on the inhale and exhale phases corresponding to the maximum extent of target motion and will be propagated to the average CT dataset for treatment planning and dose calculation. A Planning Target Volume (PTV) margin of ≥ 5 mm will be added to the ITV, to account for set-up uncertainty.

Where a 4DCT is not acquired, the target(s) will be delineated by one of the radiation oncologist investigators on the helical CT scan, who will designate the GTV/CTV. A PTV margin of ≥ 5 mm will be added to the GTV/CTV, to account for set-up uncertainty and possible motion due to respiration.

Targets must be suitable for treatment within a maximum field size of 7.5 cm x 30 cm. If this is not possible, the study participant does not meet the final inclusion criterion and will be removed from the study and will not count towards the final enrollment goal of 10 treated study participants.

A single, 250 MeV transmission proton field will be used for planning. In order to ensure acceptable coverage and minimize dose to surrounding normal tissues, gantry angle, couch angle, field size and field shape (within the field tolerance) shall be appropriately selected. Consideration of beam angles that minimize impact of motion should also be made where possible.

Since the treatment will be delivered with transmission FLASH, there will be no Bragg Peak inside the body. The relative biological effectiveness (RBE) of 1.0 will therefore apply to the transmission FLASH treatment since no correction is required for Bragg Peak.

A dose of 8 Gy in a single fraction will be prescribed to the PTV at a dose rate ≥ 40 Gy/s. This dose regimen is a standard dose and fractionation for painful bone metastases, whose efficacy has been validated in prior multi-institutional prospective randomized clinical trials [15, 16].

The volume of PTV receiving 90% of the prescribed dose shall be greater than or equal to 90%, and the dose to 10% of the PTV will not exceed 110%.

There are no published dose constraints for OARs for palliative 8 Gy RT regimens, likely since historical rates of toxicities from this low-dose palliative regimen are low. However, to be conservative, relevant maximum dose limits to a point or volume for the spinal cord, esophagus and heart, and volumetric dose constraints for the lung will be followed and are presented in Table 1 (below). Given the thoracic sites of bone metastases eligible for treatment, the expectation is that there may be some lung in the radiation field. In addition, though patients with spinal metastases in the thoracic spine are not eligible for this study, the dose limit for the spinal cord was added due to the potential for out-of-field or exit dose contribution to the spinal cord, which should be low. Dose constraints are from the recommendations in the report of AAPM Task Group 101 for single fraction stereotactic body RT [17].

**Table 1:** Normal tissue dose contraints

Tissue	Volume	Volume Max (Gy)	Max point dose (Gy)	Potential Adverse Outcome (≥ Grade 3) per TG101
Spinal Cord	< 1.2 cc	7	14	Myelitis
Lung (Right and Left)	1500 cc	7	NA-Parallel tissue	Basic Lung Function
Lung (Right and Left)	1000 cc	7.4	NA-Parallel tissue	Pneumonitis
Lung (Right and Left)	37%	8	NA-Parallel tissue	Pneumonitis
Heart/pericardium	15 cc	16	22	Pericarditis
Esophagus	5 cc	11.9	15.4	Stenosis/fistula

If more than 1 lesion is eligible for treatment with FLASH RT, the normal tissue dose limits will apply to the composite (sum) plan of all lesions treated.

The investigator will determine if the treatment plan is clinically acceptable.

A treatment plan that exceeds the dose limits in Table 1 will not be allowed. Subjects for whom clinically acceptable treatment plans cannot be created will not continue on the study (and will not undergo FLASH RT treatment) and will be replaced. The investigator will verbally communicate this to the subject, assist in making alternative arrangements for treatment, and document it on an end-of-study form.

### Radiotherapy quality assurance (QA)

Treatment plans created in the FLASH-capable Eclipse TPS 16.5 system will be imported into the ARIA Oncology Information System (Varian Medical Systems, Palo Alto). As per routine practice, daily machine QA and patient-specific QA will be performed in preparation for FLASH therapy treatments.

Machine QA includes standard daily machine QA (e.g., safety checks, output checks) as well as dose rate verification using the FLASH QA tool. The FLASH QA tool is stand-alone software that analyzes the Pencil Beam Scanning log files associated with FLASH treatment plans to verify that the FLASH-enabled ProBeam is delivering at FLASH dose rates of at least 40 Gy/s.

Patient-specific QA for study participants treated on this study protocol will be performed, for example, using film and/or ion chamber dosimetry. The patient-specific QA program is substantially similar to standard Intensity-Modulated Proton Therapy (IMPT) patient-specific QA. The patient quality assurance procedure determines whether a planar dose calculation is within a clinically acceptable tolerance of a planar dose measurement.

An RT review committee, consisting of an external group of expert radiation oncologists, will perform post-treatment review of FLASH RT plans for every third study participant.

### FLASH therapy

The present clinical study (FAST-02), like the preceding FAST-01 trial, will be carried out at an investigational site with a ProBeam Proton Therapy System (Varian Medical Systems, Palo Alto), an FDA cleared device (K133191), that utilizes a cyclotron to deliver proton radiation, and that has been modified to deliver the proton radiation at a FLASH dose rate.

The treatment plan will be transferred from ARIA to the ProBeam Proton Therapy System console for FLASH RT delivery. Image guidance will be used to verify that the target is in the correct position for treatment.

## Follow-up

A summary of study visits and activities is provided in Table 2. It will be acceptable to carry out remote follow-up visits as an alternative to in-person visits in the event of the study participant’s inability or reluctance to travel as well as for progressive subject disability. In these circumstances, photographs of the treatment site may be taken at home by caregivers, questionnaires may be completed at home as the subject’s clinical status permits, and physical evaluations may be carried out to the extent those are feasible via telehealth. A mobile nurse may also be used for home visits to carry out study activities such as physical assessments and assisting subjects with data collection when in-person visits are not possible. If remote follow-up is not possible, then an effort will be made to collect study data from a review of subject records (e.g., electronic medical records, hospice records, etc.) as available to the investigator.

**Table 2:** Schedule of activities

	Treatment Simulation to Treatment Delivery (≤ 10 business days)				Post Treatment Follow-up						
**Event**	Enrollment, baseline (1–2 weeks)	Prep for treat-ment	Prior to treatment delivery (Day 1)	Treatment delivery (Day 1)	After treatment delivery (Day 1)	Day 2 - Day 11 (daily)^1^	Day 2 (± 1 calendar day)	Day 7 (± 2 business days)	Day 15 (± 2 business days)	Month 1 (± 5 business days)	Months 2, 3, 6, 9, 12, then every 6 months
Patient screening (initial inclusion/ exclusion criteria)	X										
Informed consent	X										
Demographics	X										
Disease information	X										
CT-based simulation		X									
Treatment planning		X									
Treatment plan QA		X									
FLASH treatment delivery				X							
Questionnaire for pain flare assessment			X			X					
Questionnaire for overall pain assessment			X					X	X	X	X
Questionnaire for pain assessment at treated sites			X					X	X	X	X
Subject evaluation^2^	X		X					X	X	X	X
Review of pain medication (including steroid medications)			X			X		X	X	X	X
AE evaluation^3^					X		X^4^	X	X	X	X

^1^ Day 2-Day 11 subject evaluation: review of pain medications (including steroid medications) and pain flare assessment will be completed by telephone check-in.

^2^ Includes performance status and physical evaluation with skin photographs.

^3^ All AEs and SAEs will be captured regardless of severity or attribution.

^4^ Day 2 AE check will be carried out through a remote visit.

Outcomes will be evaluated against data collected as part of the baseline evaluation and on the day of treatment (but before start of treatment) as follows:

- Subject characteristics: date of birth, sex, ethnicity, race, history of medical comorbidities or genetic or autoimmune disorders, diagnosis date, and prior cancer-directed treatments.
- Tumor characteristics: histology, anatomic location of the original primary tumor and the anatomic location of treatment site(s), target lesion size, and radiographic appearance of the target (e.g., lytic, sclerotic).
- Baseline data specific to outcome assessments: performance status, photographs of skin and any physical findings on examination involving the skin or other normal tissue in the planned treatment site(s), and information about treatment for other bone metastases not treated as part of this study.
- Pain medications (including narcotics, gabapentin class drugs, other non-narcotic analgesics, and steroid medications).

If a study participant leaves the study for any reason before the next scheduled follow-up visit is completed, the investigator will document the reason(s) to the extent known by the investigator. In addition, the investigator will attempt to collect a final assessment of the following: patient-reported pain score (overall and specifically for treated site(s)), use of pain medications (including steroid medications), and AEs, including skin and other normal tissue toxicities.

## Outcomes

### Toxicity

AEs will be monitored from the start of treatment to the completion of the final study follow-up visit. All AEs will be collected regardless of severity or attribution and will be classified per Common Terminology Criteria for Adverse Events (CTCAE) version 5.0. The outcome of toxicities that are possibly, probably, or definitely attributed by the investigator to FLASH RT will be assessed through AE checks, photographs, and pain flare questionnaires. The AE check on Day 2 will be carried out by remote review. In some circumstances, AE checks during follow-up may be carried out using a combination of remote visits and/or records review.

Photographs of skin at the entry and exit sites of the radiation beam will be captured at AE checks to facilitate grading and attribution of any skin AEs. If the treated area(s) are readily identifiable, the photographs should encompass the entire anatomic region treated with close-up photographs of the skin in each treated area.

Pain flare (transient increase in treated site bone pain following RT treatment) will be assessed at Day 1 (baseline pain prior to treatment) and daily for the first 10 days (Day 2–11) following the treatment day using the methodology of Chow et al. [18]. This patient-reported assessment will collect the following information for each treated site: (1) worst pain over the last 24 hours and (2) patient-reported comparison of this worst pain to the worst pain in the treated site on the day of treatment. Participants’ use of pain medication will be logged.

### Efficacy

Patient-reported overall pain will be collected through the use of the brief pain inventory (BPI) short-form questionnaire. Patient-reported pain for each treated site will be assessed using a four-item subset of the BPI questionnaire [10]. These questionnaires (for overall pain and pain at the treated sites) will be completed on Day 1 (prior to treatment) and on Day 7, Day 15, Month 1, Month 2, Month 3, Month 6, Month 9, Month 12, and every 6 months thereafter. They may be collected using a combination of in person or remote visits and/or, from Day 7 onward, medical records review.

Use of pain medication will be collected and changes in pain medication use will be evaluated between baseline, during each of the first 10 days after treatment, and follow-up visits Day 7, Day 15, Month 1, Month 2, Month 3, Month 6, Month 9, Month 12, and every 6 months thereafter.

### Clinical workflow

The FLASH clinical workflow outcomes are (1) time on the treatment table and (2) occurrence of any device issues. Device issues include, but are not limited to, delays in study treatment related to the investigational device, excluding delays due to subject or facility factors not related to study treatment.

## Stopping rules

Triggering of any one of the following rules will result in a pause of enrollment by the Sponsor, followed by Sponsor and DSMB review of the study data if:

- 2 out of 6 study participants, or if 3 or more study participants, experience grade 3 or higher toxicity that is possibly, probably, or definitely related to study treatment;
- 1 subject experiences a grade 5 AE, unless probably or definitely related to the underlying disease, complications of subsequent cancer therapies, or extraneous causes;
- A major device malfunction in dose delivery occurs (as indicated by the dose monitoring system). This includes potential recordable or reportable medical events under the Ohio Department of Health classification.

The DSMB will make recommendations regarding continuation or cessation of enrollment.

## Results

Enrollment for the study is expected to take 15 months. Subjects will be on study prior to their radiation treatment simulation (approximately 1–2 weeks); during their radiation treatment simulation, treatment planning, and delivery (≤ 10 business days); and during post-treatment follow-up (until subject death or lost to follow-up). The study may run from 2.5 years to several years (which covers enrollment, treatment, follow-up, and completion of data analyses).

## Discussion

Bone metastases are a common complication of cancer. The pain associated with bone metastases can have a significant adverse impact on a patient’s quality of life. While other focal modalities are being evaluated as an alternative or supplement to RT, such as magnetic resonance image-guided high-intensity focused ultrasound (the three-armed randomized controlled ‘FURTHER’ trial) [19], external beam RT remains a current standard of care for pain management.

The FAST-02 study is designed to assess the pain relief and toxicities of treatment in patients with painful thoracic bone metastases treated with FLASH RT. It is the first clinical trial evaluating FLASH therapy for this clinical indication and for the treatment in the thorax, and only the third prospective clinical trial to be activated worldwide studying ultra-high dose-rate FLASH RT.

The US Food and Drug Administration (FDA) has authorized the clinical investigation to move forward with FLASH therapy in a study limited to 10 participants. Proton FLASH is a novel treatment strategy, used previously in only one prospective proton clinical trial (FAST-01), and the clinical data generated by this FAST-02 protocol will contribute to the assessment of the safety and efficacy of this treatment modality. Until additional data are generated on the risk of AEs from FLASH, spinal metastases (requiring direct FLASH irradiation of the spinal cord) are not eligible for treatment under this protocol. To spare normal tissue in the region of the treatment, this study will use image-guided setup for the FLASH treatment and a conformal treatment planning technique.

The data acquired in this investigation will provide clinical data to answer the question of whether FLASH therapy is safe and effective in the treatment of thoracic bone metastases. Based on the results of FAST-01, treatment is expected to be effective and well-tolerated. If the results prove to be favorable, this study will support the further exploration of FLASH therapy for broader clinical indications.

## Data Availability

N/A.

## Abbreviations

4DCT: Four-dimensional computed tomography
AE: Adverse event
SAE: Serious adverse event
CCHMC: Cincinnati Children’s Hospital Medical Center
CT: Computed tomography
CTCAE: Common Terminology Criteria for Adverse Events
DSMB: Data safety monitoring board
RT: Radiation therapy
ITV: Internal Target Volume
